# Endovascular Treatment of a Bilateral, Ruptured Angiomyolipoma in a Patient With Tuberous Sclerosis Complex

**DOI:** 10.7759/cureus.66200

**Published:** 2024-08-05

**Authors:** Maciej Mach, Karol Maciejewski, Tomasz Ostrowski, Rafał Maciąg, Michał Sajdek, Zbigniew Gałązka

**Affiliations:** 1 Department of General, Vascular, Endocrine and Transplant Surgery, Medical University of Warsaw, Warsaw, POL; 2 2nd Department of Clinical Radiology, Medical University of Warsaw, Warsaw, POL

**Keywords:** renal tumor, renal intervention, tuberous sclerosis complex, embolization, renal angiomyolipoma

## Abstract

A renal angiomyolipoma (AML) is a rare, usually benign tumor consisting of smooth muscle cells, abnormal blood vessels, and fat tissue. Although AMLs are often asymptomatic, they can present with flank pain, hematuria, and a palpable mass in the abdomen. A significant complication involves rupture and hemorrhage into the retroperitoneal cavity, which can be life-threatening. The treatment approach has evolved from surgical removal to more conservative management, such as nephron-sparing embolization and mammalian target of rapamycin (mTOR) inhibitors for tuberous sclerosis complex (TSC)-associated AML. In March 2024, a 36-year-old female patient diagnosed with TSC was admitted to our department and underwent several endovascular embolizations after a life-threatening hemorrhage from a ruptured multilocular AML. The treatment was successful, with complete exclusion of the AMLs from circulation and without any complications during the postoperative period. This case emphasizes the effectiveness of selective arterial embolization using the Onyx liquid embolic system in managing AMLs and highlights the importance of preserving renal function. Methods used in AML diagnosis include ultrasound and computed tomography scans, with magnetic resonance imaging and biopsy recommended in difficult cases. Treatment depends on aspects such as tumor size, symptoms, and patient's general condition, with options ranging from active surveillance for small, asymptomatic AMLs to more invasive procedures for larger, symptomatic tumors. The main goal is to minimize symptoms and preserve renal function.

## Introduction

A renal angiomyolipoma (AML) is a relatively rare tumor, with a prevalence of 0.2% in the general population, commonly consisting of smooth muscle cells, abnormal blood vessels, and fat tissue in different proportions [1]. AMLs are typically regarded as benign, with a very low risk of becoming malignant and developing aggressive behavior [2]. Patients with renal AML often experience no symptoms, nevertheless, they may also present with flank pain, gross hematuria, palpable mass, and bleeding in the retroperitoneum. The main and high mortality complication of renal AMLs is their rupture and hemorrhage into the retroperitoneal cavity [3]. The management of AML has evolved over the years. Initially, renal AMLs were surgically removed, but later a more conservative approach emerged, involving nephron-sparing embolization. Eventually, the mammalian target of rapamycin (mTOR) inhibitors proved effective in halting tuberous sclerosis complex (TSC)-associated renal AMLs [4]. We present a case of a young woman diagnosed with numerous AMLs in both kidneys who underwent multiple endovascular embolizations.

## Case presentation

In March 2024, a 36-year-old female patient diagnosed with TSC was admitted to our clinic for preventive embolization of a nodular lesion in the right kidney. Her prior medical history revealed cholelithiasis, resection of a benign thyroid tumor, and epilepsy in childhood. Moreover, significant bleeding occurred due to a ruptured right renal AML in January 2024. At that time, the patient was treated conservatively with the transfusion of two units of packed red blood cells and two units of plasma. On general examination, the blood pressure was 130/85 mm Hg with a regular pulse rate of 78 beats per minute. The abdomen was soft and painless, and there was no peripheral edema. The laboratory results indicated mild anemia with preserved kidney function (Table 1). 

**Table 1:** Laboratory parameters on admission eGFR: Estimated Glomerular Filtration Rate

Parameter	Result	Reference Range
Hemoglobin	11.6 g/dl	12-16 g/dl (for women)
Hematocrit	34%	36-46% (for women)
Platelets	185 x 10³/µl	150-450 x 10³/µl
Creatinine	0.71 mg/dl	0.5-1.1 mg/dl (for women)
eGFR	110 ml/min/1.73 m²	>90 ml/min/1.73 m²

**Figure 1:**
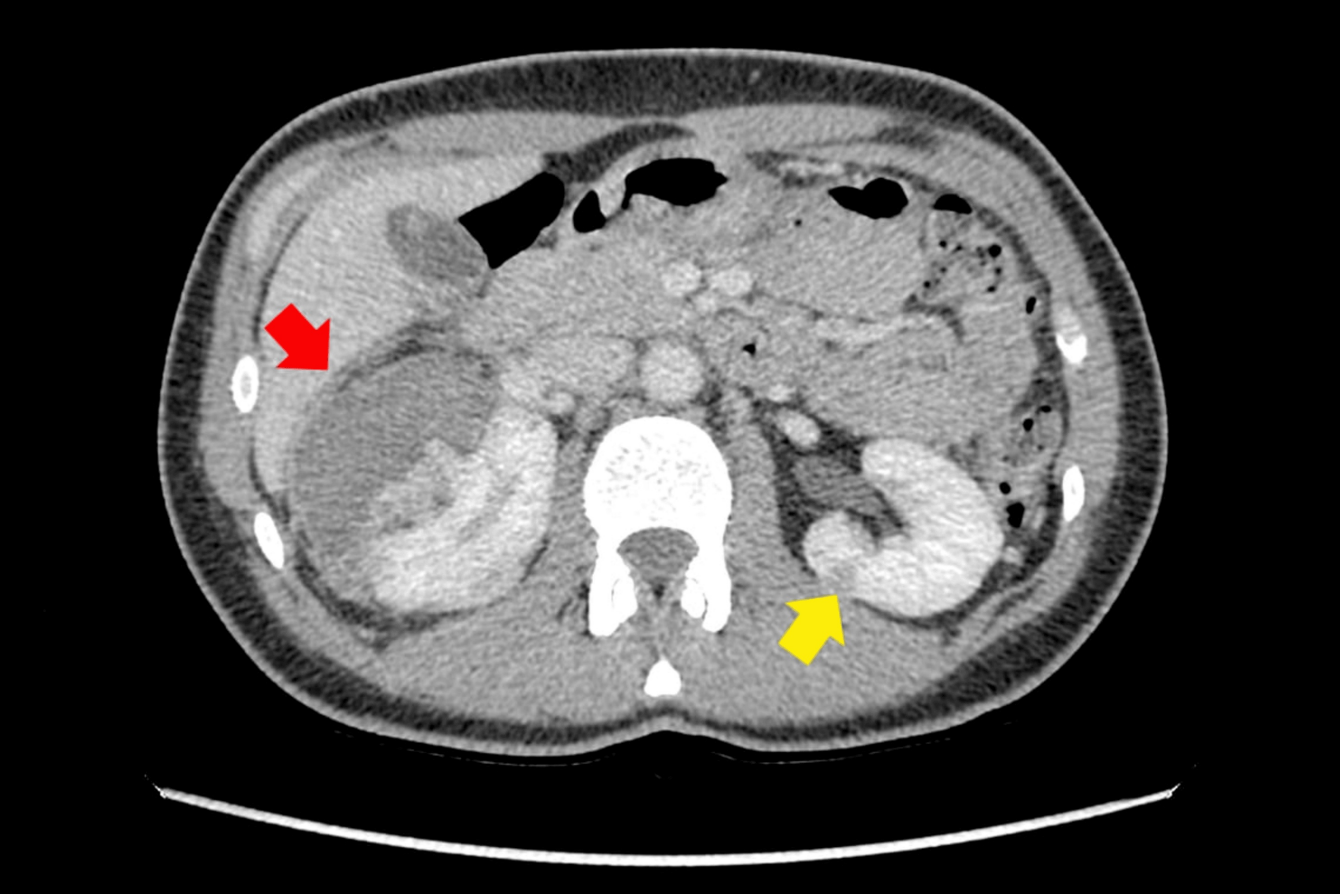
Multiple focal lesions in the right kidney (red arrow) and left kidney (yellow arrow)

Computed tomography angiography revealed multiple focal AML lesions in both kidneys (Figure 1).

**Figure 2:**
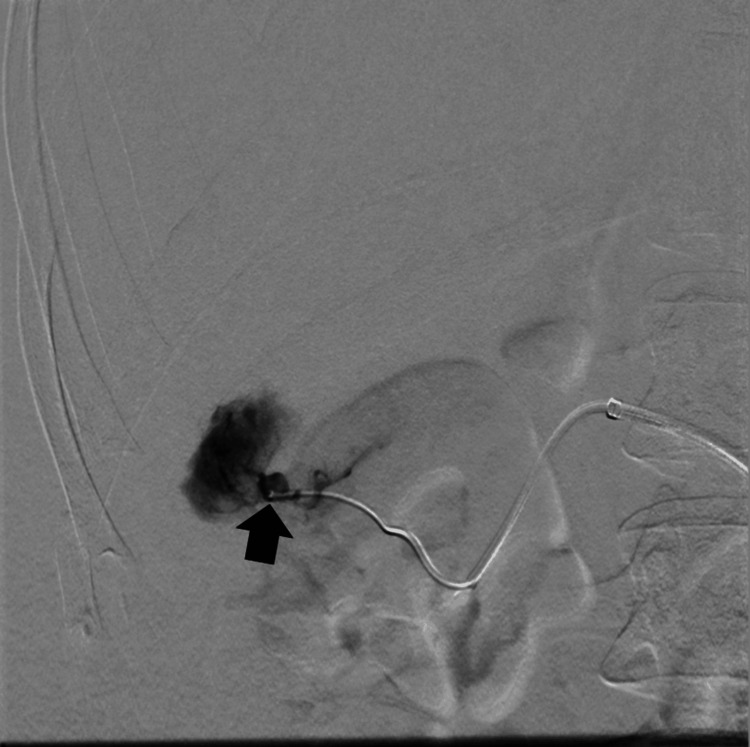
A ruptured lesion in the right kidney (black arrow)

**Figure 3:**
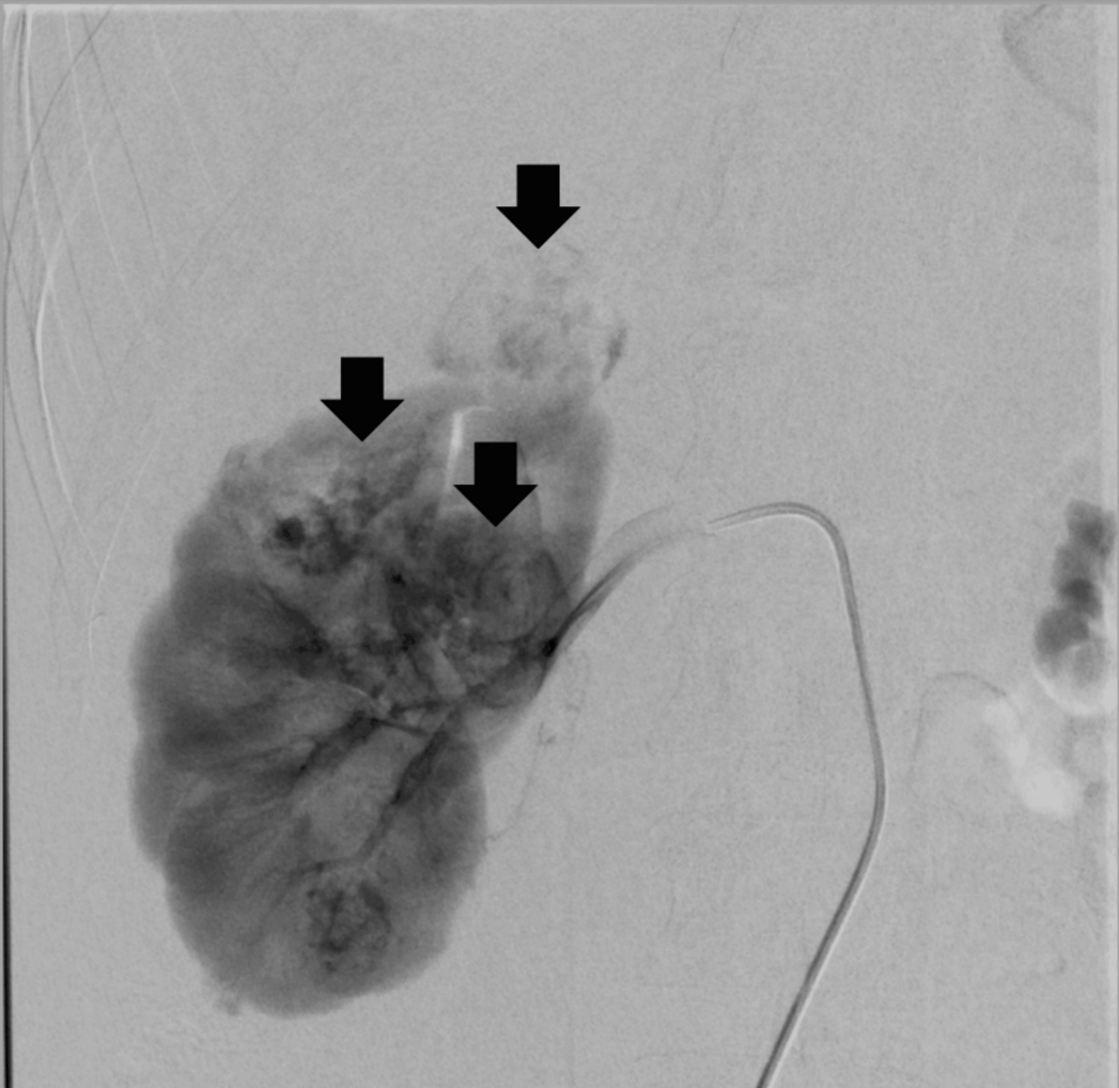
AML lesions in the right kidney during angiography (black arrows)

Due to the patient's young age, the potential for pregnancy, and the minimally invasive nature of the procedure, the decision was made to proceed with the surgery. In March 2024 through right common femoral artery endovascular access, selective embolization of the previously ruptured lesion (Figure 2) and three additional lesions in the right kidney parenchyma (Figure 3) was performed using three ampoules of Onyx-20 liquid embolic agent.

**Figure 4:**
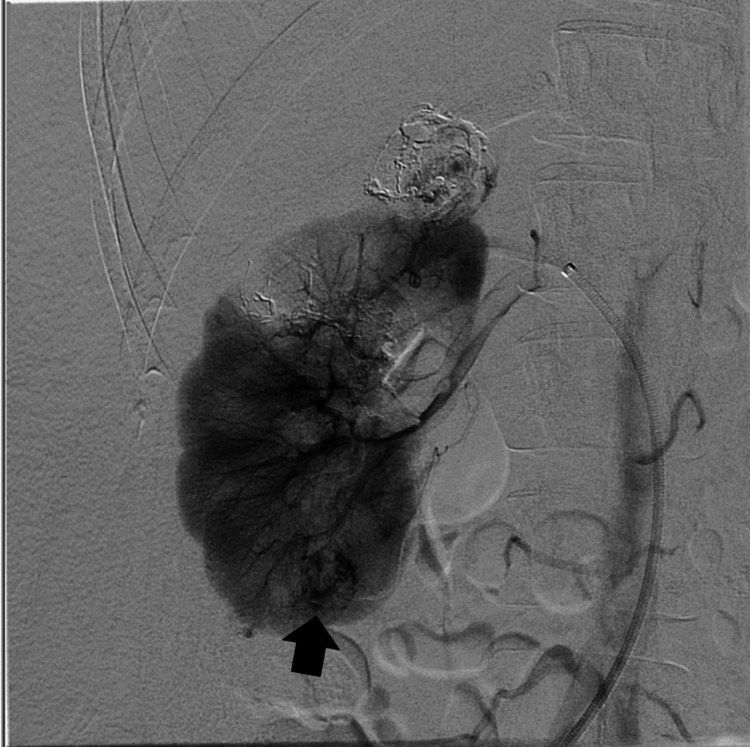
Right kidney nephrogram after embolization of three AML lesions and a visible lower pole AML lesion (black arrow) AML: Angiomyolipoma

A small pseudoaneurysm was visible within the post-hemorrhagic lesion, which was closed during tumor embolization. Control angiography revealed partial filling of a fragment of the tumor located in the upper pole of the right kidney (incomplete embolization) (Figure 4).

**Figure 5:**
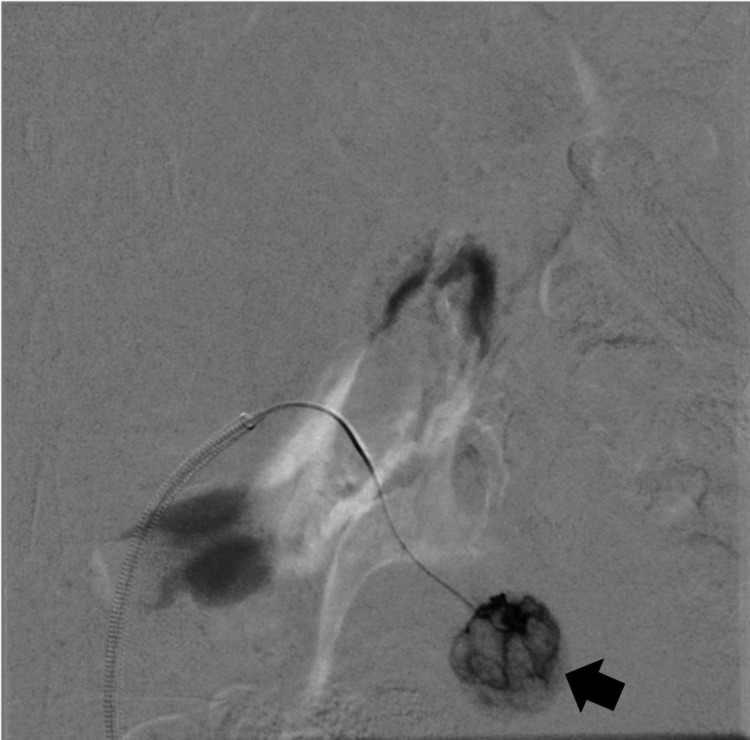
AML lesion in the left kidney during angiography (black arrow) AML: Angiomyolipoma

Additionally, single focal lesions were visible in the lower parts of both kidneys (Figure 4, 5), classified for possible future embolization, the size of the lesion in the lower pole of the right kidney was approximately 1.8 cm, so embolization was not pursued. The arterial endovascular access site in the right inguinal area was secured using the FEMOSEAL occlusion system with an additional pressure dressing.

**Figure 6:**
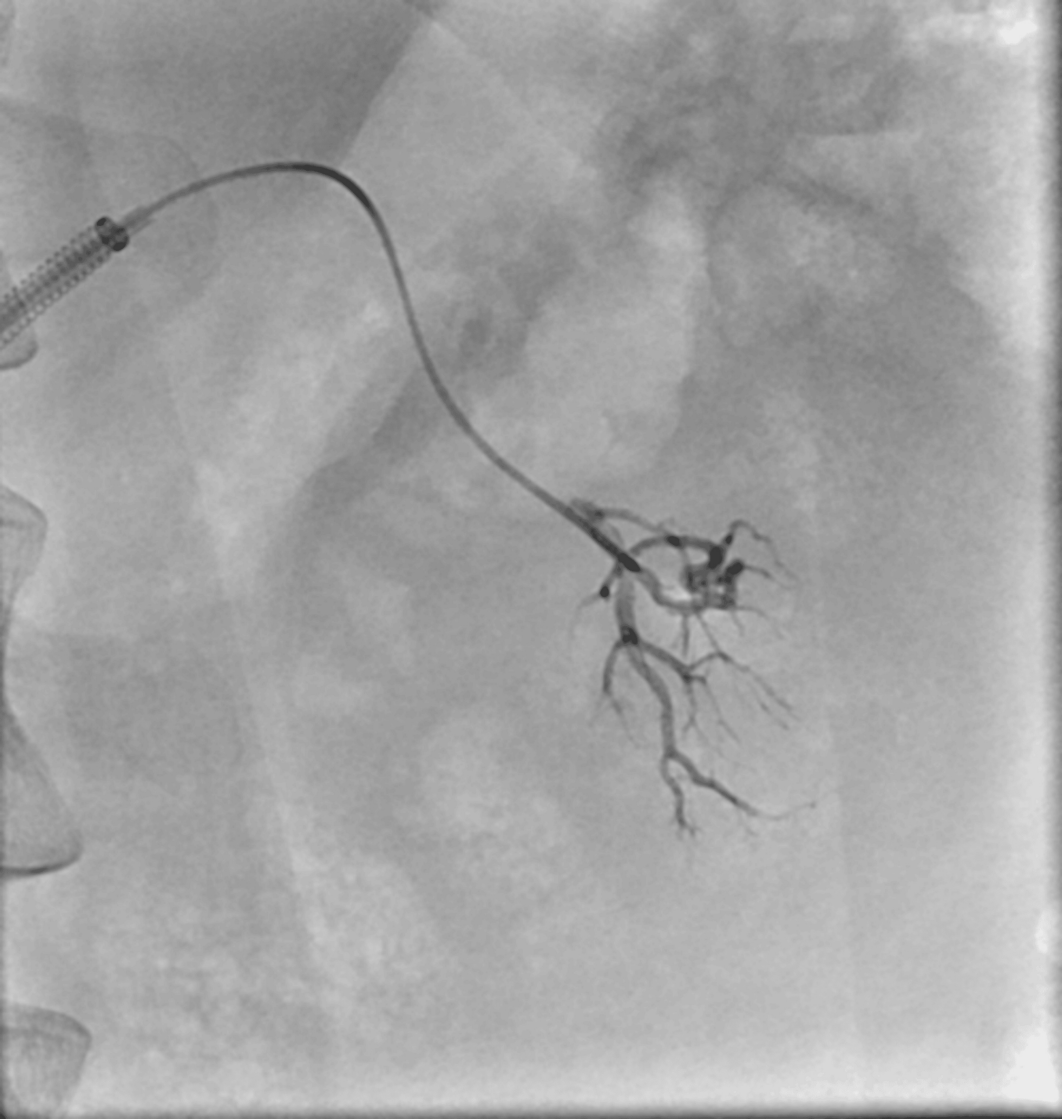
Embolization of the AML lesion in the lower pole of the left kidney using Onyx-18 AML: Angiomyolipoma

**Figure 7:**
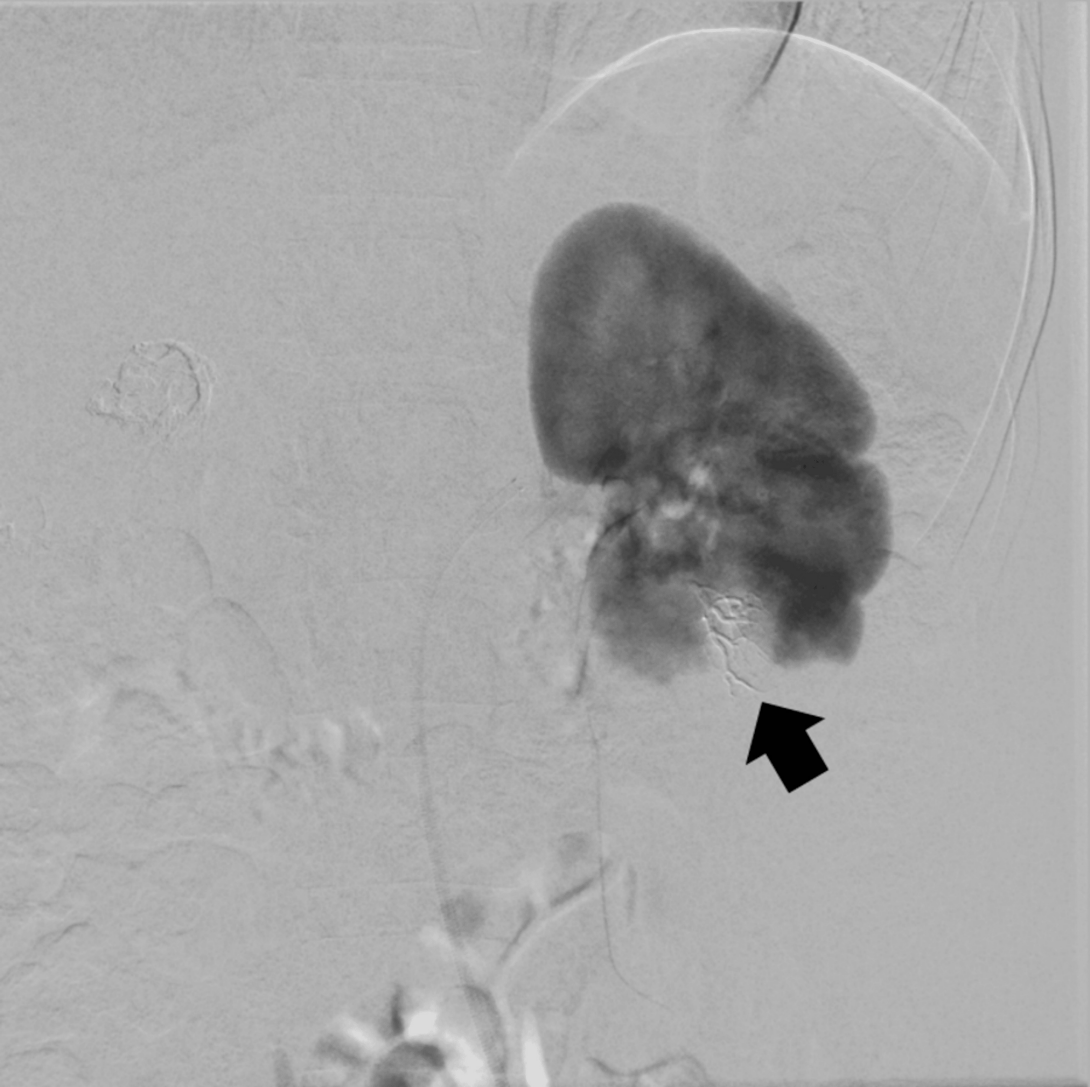
Embolized lesion in the lower pole of the left kidney (black arrow)

During the procedure, 5,000 units of heparin were administered. The postoperative period was uncomplicated. The puncture site of the right common femoral artery healed properly and a pulse was palpable. The patient was discharged home on the third postoperative day. In May 2024, the patient underwent the next stage of the treatment. Under local anesthesia, via the left common femoral artery selective embolization of an AML lesion in the lower left renal pole was performed (Figures 6, 7).

**Figure 8:**
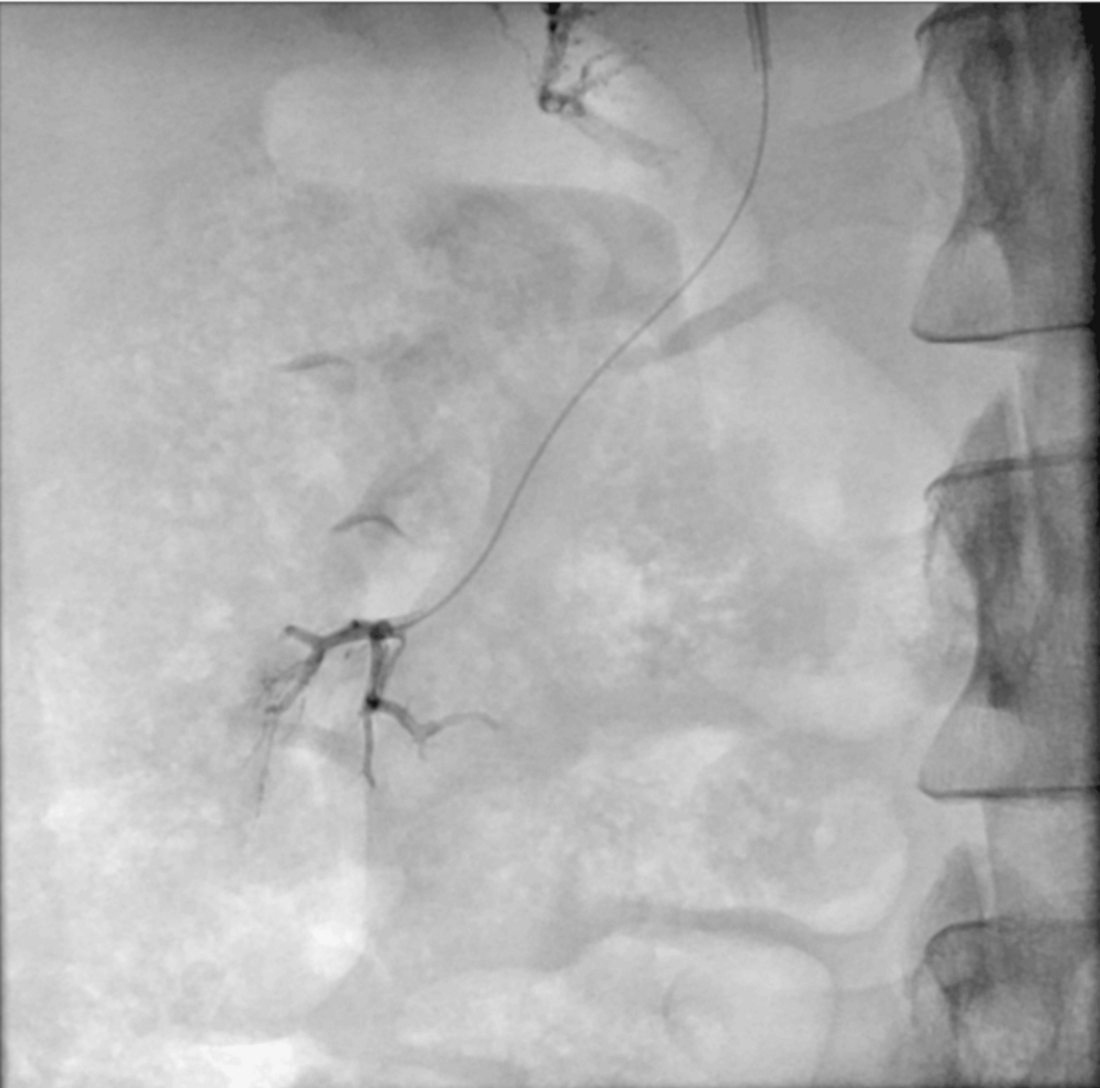
Embolization of the AML lesion in the lower pole of the right kidney using Onyx-18 AML: Angiomyolipoma

In the subsequent phase, lesions in the right kidney's lower pole were embolized with the utilization of two ampoules of the Onyx-18 liquid embolic system (Figure 8). 

**Figure 9:**
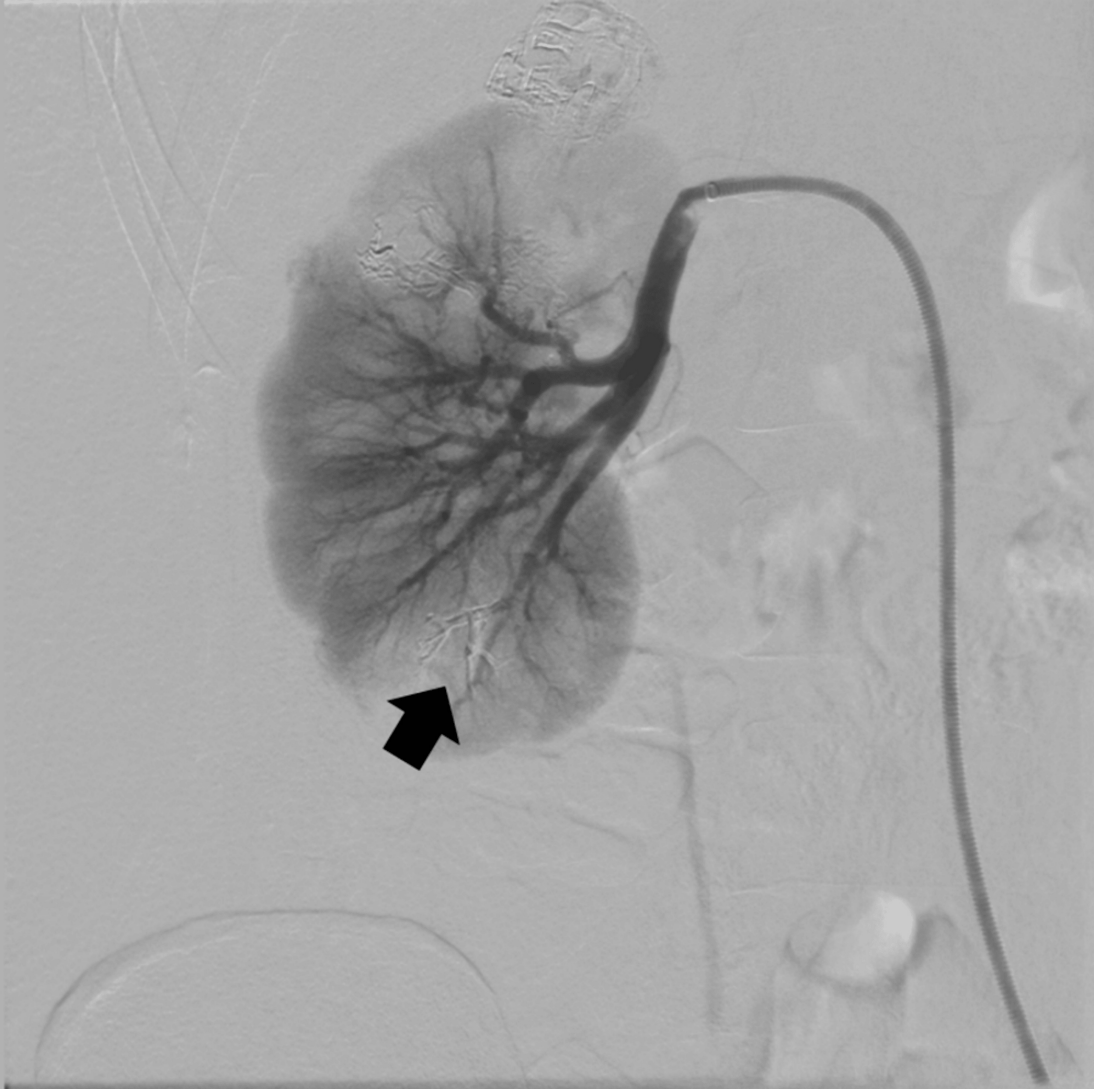
Embolized lesion in the lower pole of the right kidney (black arrow)

Vascular access was secured with the FEMOSEAL occlusion system. Control angiography revealed complete exclusion of AMLs from circulation (Figure 9).

## Discussion

Etiology and epidemiology

Classified as a perivascular epithelioid cell tumor, the AML is one of the most frequent benign mesenchymal renal neoplasms, predominantly found in females rather than males [5]. Renal AMLs can be divided into two main categories: sporadic and syndromic AMLs. The sporadic form includes triphasic benign AMLs, which encompasses classic AMLs and fat-poor AMLs, as well as epithelioid AMLs. The syndromic form includes AMLs associated with TSC and lymphangioleiomyomatosis [6]. TSC-AMLs, found in younger age, are prone to more rapid growth, a higher risk of bleeding, a greater chance of malignancy, and multilocular localization [5]. Sporadic AMLs, most commonly found in patients between 50 and 60 years old, are characterized by slow expansion and unilateral localization. The age and gender of our patient align with the higher prevalence of renal AMLs in younger females with TSC.

Clinical manifestation

Approximately 80% of AML cases remain asymptomatic and are commonly found accidentally, while the remaining 20% usually present typical symptoms: abdominal pain, hematuria, and palpable mass in the abdomen [7]. The main indication of AML is non-traumatic, spontaneous, life-threatening bleeding into the retroperitoneal cavity, commonly known as Wunderlich’s syndrome, often accompanied by Lenk's triad: acute flank pain, flank mass, and hypovolemic shock [8]. AML bleeding and rupture are highly correlated with tumor vascularization, its size, and the existence of intratumoral aneurysms larger than 0.5 cm [9]. The patient's presentation with a ruptured right renal AML is consistent with the main complication observed in TSC-associated AMLs, which due to their vascular nature are prone to bleeding.

Diagnostics

Technologies most frequently used to diagnose and choose the appropriate treatment for renal masses include ultrasound (US), computed tomography (CT), and magnetic resonance imaging (MRI) [10]. On US, because of the presence of macroscopic fat, the AML resembles a hyperechoic lesion that cannot be differentiated from other renal tumors [11]. CT is commonly the first imaging method utilized, frequently conducted for other purposes, and can be sufficient for adequate diagnosis [12]. In CT scans, AML typically appears as distinct renal cortical nodules with well-defined borders, displaying a pyramidal and outward growth pattern, containing varying amounts of fat (ranging from -10 to -120 Hounsfield Units) and regions of soft density that become more pronounced after contrast administration [13]. MRI is recommended in difficult cases, however, aspects such as the absence of radiation and additional information supplied by specific sequences make it a convincing alternative to CT [10]. A fat-rich AML, on T2-weighted MRI scans, is usually hyperintense in comparison to renal parenchyma. Fat-poor is slightly hypointense compared with renal parenchyma; however, the distribution of fat might affect the features of imaging. In cases of fat-invisible AML, the lesion is homogeneously hypointense relative to renal parenchyma and the signal intensity might be caused by the main component of the lesion, which is muscle [14]. Another useful tool is renal biopsy. Indications for renal biopsy include intratumoral cysts, solitary fat-poor AMLs, intratumoral calcifications, or uncertain MRI images. Biopsy may be particularly helpful in patients with coexisting AMLs and atypical lesions to assess tumor characteristics [15]. Diagnostic tools applied to our patient provided detailed visualization of the lesions. The findings of multiple focal AMLs are typical in TSC patients.

Treatment

When choosing the treatment option, factors such as clinical presentation, the number and size of the tumor, existing medical conditions, renal function, and the risk of malignancy should be taken into account. The tumor’s diameter primarily determines the treatment choice; an AML smaller than 4 cm usually presents no symptoms and the patient can remain under active surveillance [16]. When the tumor exceeds 4 cm and the symptoms become persistent, the therapeutic goal should be focused on preserving renal function to the maximum achievable degree, involving mesenchymal-sparing treatment such as partial nephrectomy, selective arterial embolization (SAE), ablation, and mTOR inhibitors in patients with TSC-associated AMLs [11]. SAE has many advantages over surgical treatment. This procedure leads to less physical trauma and a quicker recovery period. Less invasive treatment is also suitable for patients with high surgical risk caused by other comorbidities. It helps to significantly decrease the risk of severe complications [7]. The decision to use SAE in our patient is consistent with the current approach for symptomatic or complicated AMLs.

Unique aspects of this case include quick progression from presentation to multiple staged embolizations within a short period of time. This highlights the importance of timely intervention in effective management of multiple lesions and the prevention of recurrent hemorrhage. Another important aspect is consideration of our patient's young age and potential for pregnancy in the treatment planning process. This is particularly significant, as it ensures that applied treatment options address not only immediate clinical needs but also long-term reproductive health.

## Conclusions

A renal AML is an uncommon benign tumor, usually not causing clinical manifestations, and is diagnosed accidentally. The risk of AML rupture is believed to increase with the tumor’s size. The management of this tumor relies on the patient's clinical symptoms and should be customized to each individual, ranging from close surveillance to more invasive interventions.
